# A Multiscale Polyp Detection Approach for GI Tract Images Based on Improved DenseNet and Single-Shot Multibox Detector

**DOI:** 10.3390/diagnostics13040733

**Published:** 2023-02-15

**Authors:** Meryem Souaidi, Samira Lafraxo, Zakaria Kerkaou, Mohamed El Ansari, Lahcen Koutti

**Affiliations:** 1 LABSIV, Computer Science, Faculty of Sciences, University Ibn Zohr, Agadir 80000, Morocco; 2 Informatics and Applications Laboratory, Computer Science Department, Faculty of Sciences, University of Moulay Ismail, Meknès 50070, Morocco

**Keywords:** polyp, wireless capsule endoscopy images (WCE), single-shot multibox detector (SSD), image augmentation, multiscale DenseNet

## Abstract

Small bowel polyps exhibit variations related to color, shape, morphology, texture, and size, as well as to the presence of artifacts, irregular polyp borders, and the low illumination condition inside the gastrointestinal GI tract. Recently, researchers developed many highly accurate polyp detection models based on one-stage or two-stage object detector algorithms for wireless capsule endoscopy (WCE) and colonoscopy images. However, their implementation requires a high computational power and memory resources, thus sacrificing speed for an improvement in precision. Although the single-shot multibox detector (SSD) proves its effectiveness in many medical imaging applications, its weak detection ability for small polyp regions persists due to the lack of information complementary between features of low- and high-level layers. The aim is to consecutively reuse feature maps between layers of the original SSD network. In this paper, we propose an innovative SSD model based on a redesigned version of a dense convolutional network (DenseNet) which emphasizes multiscale pyramidal feature maps interdependence called DC-SSDNet (densely connected single-shot multibox detector). The original backbone network VGG-16 of the SSD is replaced with a modified version of DenseNet. The DenseNet-46 front stem is improved to extract highly typical characteristics and contextual information, which improves the model’s feature extraction ability. The DC-SSDNet architecture compresses unnecessary convolution layers of each dense block to reduce the CNN model complexity. Experimental results showed a remarkable improvement in the proposed DC-SSDNet to detect small polyp regions achieving an mAP of 93.96%, F1-score of 90.7%, and requiring less computational time.

## 1. Introduction

**Figure 1:**
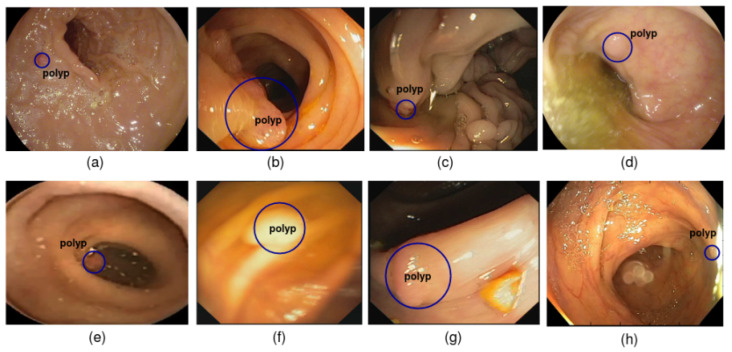
Examples of polyp image artifacts used in the current study. (**a**) Bubbles; (**b**) images with medical instruments; (**c**) white liquid and specular reflections; (**d**) cloudy liquid and specular reflections; (**e**) debris; (**f**) blurry images caused by different factors; (**g**) bile and specularity; (**h**) low contrast between polyp region and normal tissues.

Recently, small bowel tumors have been the third leading cause of death in the word. Adenomatous polyps formed by glandular tissue are considered as one of the most common cases of colorectal cancer. Contrary to hyperplastic polyps, which have no malignant potential, adenomas are considered precancerous and can transform into malignant structures despite being benign tumors. The prevalence of this disease is expected to rise in the coming years [1]. For that reason, the endoscopic removal of benign and early malignant polyp regions in the GI tract in their early stage is required. Thus, doctors need a full direct visualization of the GI tract [2]. Wireless capsule endoscopy (WCE) is an advanced tool that revolutionizes the diagnosis technology [3]. It provides a feasible noninvasive method for detecting the entire gastrointestinal (GI) tract without pain and sedation compared with traditional colonoscopies. However, its short working time, low image resolution, and low frame rates restrict its wide application. In fact, the large quantity of data produced per examination per patient (approximation 55,000 images) is a laborious task for physicians to accurately locate the polyp regions in each WCE frame. An automated tumor detection technique is required to relieve specialists of the time-consuming task of reviewing the whole video before making a diagnosis. The presence of artifacts and complex characteristics (e.g., texture, shape, size, and morphology) inside the GI tract may hinder the detection process of polyp regions, as shown in Figure 1. As a result, small polyp regions are invisible to the naked eye, preventing doctors from identifying suspicious areas and manually locating polyp regions in each WCE frame. With the rise of artificial intelligence, deep learning (DL) frameworks, as opposed to handcrafted methods, have widely been investigated in medical image analysis due to their superior performance in image classification [4,5,6]. Many CAD systems have been proposed for polyp detection purposes to assist endoscopists by providing an automated tool that acquires some knowledge without requiring their physical attendance [7]. Therefore, they help clinicians to correctly determine the polyp frame’s ground truth and make the correct decision by reducing human error. Some manuscripts aimed to automatically detect and localize polyp regions on both colonoscopy and capsule endoscopic images [8,9,10]. The lack of public and annotated datasets for polyp detection purpose is a common issue in the field. However, researchers use their own dataset, and the results may lack of subjectivity. According to their initiative, other studies on colonoscopy have used public data sets (e.g., the MICCAI 2015 subchallenge on automatic polyp detection in colonoscopy). Based on their architecture, preexisting cross-domain image object detectors are split into two categories: one-stage detectors, such as YOLO [11], SSD [12], etc., and two-stage detectors (R-CNNs [13] and their numerous variations (Faster R-CNN [14], R-FCN [15], etc.)). One-stage detectors are generally faster but less accurate. Even though speed-focused object detection research in medical image analysis runs in real time on high-end GPUs, a trade-off between precision and speed prevents SSD models from detecting small objects quickly. Even though it succeeds in keeping location information in shallow/deeper layers of the network, the SSD model’s detector fails to preserve semantic information for small polyp regions. Two-stage detectors are typically more accurate but slower, and using a fixed receptive field limits deep learning’s practical application in detecting small polyp regions. The main motivation of this work is to improve the performance of the polyp detection task in term of mean average precision (mAP) with less computational cost, using more powerful deep learning frameworks. Thence, a new SSD model is redesigned based on a modified version of DenseNet. The original backbone network, VGG16, is replaced by DenseNet-46, which can address the issue of high computational runs and overfitting during the optimization process. The redesigned DenseNet framework significantly reduces the number of model parameters while improving the backbone network’s feature extraction capability. It can also capture more target information than VGG networks. The unnecessary convolution layers of each dense block are compressed to get significant increases in performance without increasing network complexity. The DenseNet-46 design reduces the number of layers used to speed up the run time while gaining significant precision, and the front stem is improved to enable the extraction of more powerful contextual information. Inspired by the DenseNet-S-32-1 [16], this manuscript presents a densely connected single-shot multibox detector (DC-SSDNet) for detecting small polyp regions in WCE and colonoscopy frames. To reach the main target, the proposed network takes advantage of the power architectural design of dense blocks to create an ultimate architecture that serves as the backbone of the SSD detector. It changes the traditional procedure of the shallow part of the VGG16 network’s alternating convolutional and pooling layers with a couple of dense blocks and transition blocks. The DenseNet architecture tends to be very deep, and an effective DenseNet compression is essential for reaping all the benefits of dense blocks, consistent with the VGG16 architecture, to target the small polyp detection while retaining computational efficiency. As a result, an entire extraction of polyp patterns of various sizes within the same layer is possible while avoiding heavy parameter redundancy. The strategy of altering dense blocks and transition blocks is aimed at incorporating contextual and semantic information into deep networks and construct a multiscale feature map. Inspired by stacking-based models, the proposal adopts the SSD network structure [12]. The backbone pyramidal network is modified to reuse missed information. Moreover, we propose a new DenseNet network to optimize the SSD network’s ability to improve small object feature extraction in the shallower network and to overcome scale variation limitations in detecting small polyp regions. Properly tuning the hyperparameters of the proposed DC-SSDNet, our experiments yield a higher mAP than the conventional SSD, with gains of 16.76, 17.74, and 16.74 points on the WCE, CVC-ClinicDB, and Etis-Larib datasets, respectively, with an enhancement of the speed–precision trade-off for the detection of small polyp regions.

The following are the primary contributions of this study:

1. The application of a modified version of DenseNet called DenseNet-46 as a backbone and smoothly adapted to the SSD detector to improve its ability for small polyp detection.
2. Based on the inception v4 stem part, the backbone DenseNet-46 front stem is improved, allowing the extraction of highly relevant features and contextual information.
3. To capture enough patterns and representative information, we increased the filter numbers of the first convolution layers in the stem part of the DenseNet-46 backbone from 32 to 64.
4. We omitted the unnecessary convolution layers of each dense block of the DenseNet-46 backbone to reduce the DC-SSDNet model’s complexity and to achieve a faster speed while preserving a lesser computation time.
5. DC-SSDNet adds a couple of new dense and transition blocks to match the structure of SSD that detects targets in images using a single deep neural network.
6. DC-SSDNet introduces additional convolution layers to the multiscale feature pyramid, which is consistent with the traditional SSD.
7. The proposed model is trained from scratch.
8. We conducted several experiments on three well-known datasets in the field (WCE, CVC-ClinicDB, and Etis-Larib) to verify the DC-SSDNet model’s effectiveness for a fair comparison with previously published methods of the literature.
9. This manuscript provides a thorough examination of the benefits and drawbacks of the proposed framework.

The rest of this paper is structured as follows: Section 2 contains references to related works. The proposed DC-SSDNet model is presented in detail in Section 3. The experimental results are reported and compared to those of other models in Section 4. Finally, Section 5 discusses the conclusions.

## 2. Literature Review

WCE images exhibit great variations in terms of size, shape, and morphology making the automated polyp detection process more difficult. Handcrafted features have been widely investigated, showing a significant progress for gastrointestinal classification tasks. A previous study [17] presented a model-based pyramid histogram for polyp classification using T-CWT and gamma. The major problem is the misunderstanding of biological mechanisms. They rely heavily on prior knowledge and have limited generalizability. Thus, handcrafted methods encode only a portion of the image and ignore the intrinsic data of the whole frame. They use low-level features created manually to describe the structures of regions that are not sufficiently robust to be applied for automatic polyp detection. Recently, the development of deep learning and powerful computing devices made the realization of deep CNNs (DCNNs) feasible. Several attempts have been made to use existing deep-learning frameworks to classify colonoscopy polyp abnormalities: VGGNet repeatedly stacked 3 × 3 convolution layers and 2 × 2 pooling layers to reach a maximum depth of 19 layers; GoogleNet [18] increased both the network depth and the width to enhance the feature representation by parallelly performing multiple convolution and pooling operations at each layer; and ResNet [19]. However, the vanishing-gradient problem always persists when training DCNNs. Inspired by the end-to-end frameworks for the polyp recognition task, Yuan et al. [16] used the most recent DenseNet model in 2019 as the basic model to directly calculate representative features from image information rather than using low-level handcrafted features to characterize the WCE image. Thus, they improved feature propagation through dense connections and significantly reduced the number of tuning while maintaining a high performance. The main proposal was to localize polyps in WCE/colonoscopy images by drawing a bounding box around the emphasis region, whereas polyp classification was a localization interstage conducted directly. In general, CNN-based methods are divided into two categories: the two-stage algorithms that generate region proposals as a first step and then classify them into different object categories (e.g., Faster R-CNN [20]), and the one-stage algorithms based on regression (e.g., YOLO [21] and SSD [22]). The Faster R-CNN [20] and R-FCN [23]) proposed anchors for different scales using a one-scale feature map. However, they failed to detect multiscale objects of small sizes. The FPN [24] and DSSD [25] methods used bottom-up and top-down frameworks, respectively; however, using the layer-by-layer feature map fusion results slowed the detection process. As a solution, the conventional SSD [12] made predictions by utilizing the feature of shallower layers and scaling them from the bottom to the top to generate a new pyramidal feature map. In this context, a two-stage framework based on deep learning was presented by Jia et al. [26] for automatic polyp recognition in colonoscopy images. The authors of [27] presented a modified version of the mask R-CNN model for performing polyp detection and for segmentation purposes. TASHK et al. [28] proposed an improved version of the CNN algorithm based on DRLSE to automatically locate polyps within a frame. However, two-stage algorithms sacrifice speed for a high-performing localization and object recognition performance. Thus, they hardly meet the real-time requirements of polyp detection. Oppositely, the one-stage algorithms achieve a high inference speed by ignoring the region proposal step and using the predicted boxes directly from the input images. To detect polyps in colonoscopy videos, Liu et al. [29] investigated the potential of the ResNet50 and VGG16 frameworks used as a backbone to propose a new architecture-based single-shot detector (SSD). However, the traditional SSD using the VGG16 network as a backbone repeatedly stacks convolution and pooling layers, and its feature extraction ability is inefficient due to its use of only 1×1 and 3×3 convolution kernels. Improving the polyp detection process necessitates a robust backbone (such as DenseNets [30]). Regarding the advantages of you only look once (YOLO) algorithms in real-time detection speed at the expense of precision, they eliminate a preprocessing step to obtain an ROI and abandon the process of proposal generation. Misawa et al. [31] presented a YOLOv3-based polyp detection system that achieved real-time detection with greater than 90% sensitivity and specificity. However, the spatial constraints of the algorithm limited it to perform with small regions within the image. Researchers made many efforts based on SSD algorithms to tackle the limitations of small polyp detection and localization. In this context, the authors of [32] proposed a rainbow SSD-based method. It applied a simple concatenation and deconvolution operation of feature maps produced from SSD layers. Zhang et al. [33] presented an SSD-GPNet-model-based SSD network by combining feature maps of the low level with the deconvolution of high-level feature maps. However, adding deconvolution layers increased the computational complexity of the SSD architectures at the expense of speed, even if it improved the performance of small polyp region detection. Regarding the success of DenseNet frameworks in many fields, Zhai et al. [16] presented an improved SSD network that used the residual prediction block and switched the network backbone to DenseNet. They then used a multiscale feature layer fusion mechanism to reinforce the relationships between the levels in the feature pyramid. However, it exhibited a decrease in detection speed by using a complex network and adding complicated feature fusion modules. To extract more semantic information while keeping the detection speed constant, we propose a densely connected single-shot detector called DC-SSDNet. The characteristic ramification of the feature maps within the same block would become quite beneficial if the purpose was to reuse them to perform stacked dense and transition blocks with the capacity of the SSD network to be more compact while going deeper. A typical compression method of a network’s feature maps is to remove unnecessary layers by reducing parameter dimensions. Concretely, there is no need for feature extraction enhancement using a lightweight feature fusion of shallower and deeper layers due to the power of dense networks for capturing more representative patterns and reassembling contextual and semantic information. Indeed, meeting the requirements of a detection process involves the reduction of the model complexity while preserving a high detection speed. Thus, clinical applications require a real-time detection and promising precision [34].

## 3. Proposed Method

**Figure 2:**
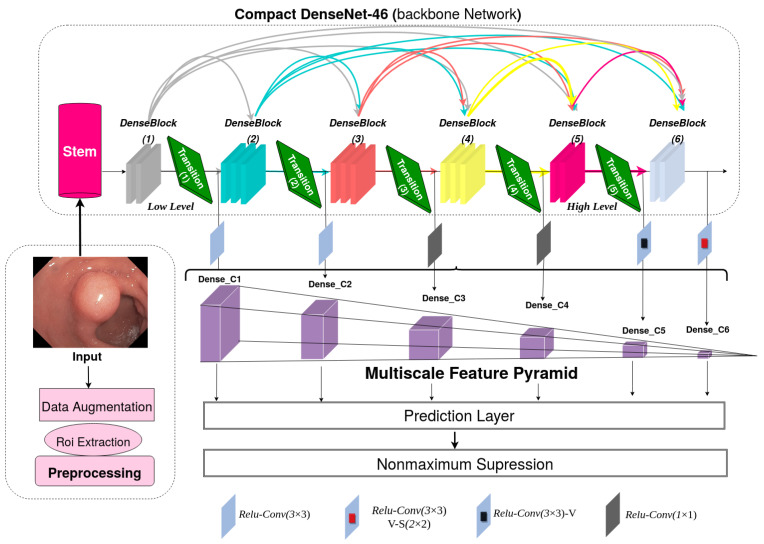
Architecture of the proposed densely connected single shot multibox detector (DC-SSDNet).

Figure 2 depicts the network structure of DC-SSDNet for the polyp detection task. A redesigned SSD-detector-based compact DenseNet-46 network used as a backbone is proposed to strengthen the network detection ability. The conventional SSD with the VGG16 as a backbone fails to preserve contextual information in the multilayer transmission process. Therefore, unbalanced feature maps appear at each layer. To reuse the rich object information of low-level layers and incorporate it with high-level layers, DC-SSDNet splits the network structure into two sections: a compact version of DenseNet (a DenseNet-46 network as a backbone) for feature reuse and extraction and the front-end network to perform multiscale object detection. Firstly, a preprocessing step is performed to remove the black regions in the WCE images and keep only useful information. Then, a data augmentation strategy is investigated to handle overfitting in deep learning models due to the data insufficiency problem. The model input size is 299×299. For object classification and location regression, the conventional SSD model selects VGG-16 layers Conv4_3 and Conv_7 and adds new ones Conv8_2, Conv9_2, Conv10_2, and Conv11_2. Consistent with the SSD network, we construct a multiscale feature pyramid under the premise of maximizing the use of synthetic information from all feature layers and improving precision without sacrificing speed. To improve the model’s detection ability, we use our own multiscale feature pyramid Dense_C1, Dense_C2, Dense_C3, Dense_C4, Dense_C5, and Dense_C6. The main parts of the DC-SSDNet detector are described in detail below.

### 3.1. Compact DenseNet-46

A deep CNN uses many repeated convolution layers resulting in the bottom-level features being destroyed. Many medical imaging applications utilize DenseNet to improve precision caused by the vanishing gradient in high-level neural networks. DenseNet reuses some not-useful redundant information to concatenate the high-level features with the residual sparse low-level ones, even if it does not propagate them effectively. In this paper, we propose a compact DenseNet-46 as a backbone network of DC-SSDNet by applying certain modifications to DenseNet. Table 1 depicts the main structure based on the number of layers. DenseNet-46 use the feature maps from all previous layers as input in the next layer to alleviate the lack of region location information of high-level features, as depicted in Table 1. Despite the success of deep DenseNets applications in many fields, we believe that using this network directly as an ultimate SSD backbone is probably not going to be an effective solution for small polyp detection. Compared to other deep DenseNet versions, the compact DenseNet-46 compresses some repeated dense connections in each dense block to allow fewer feature propagations and reduce the destruction of low-level features. Compressing each block’s unnecessary convolution layers, the compact DenseNet-46 avoids generating more redundant information, preventing the small polyp’s features from being submerged, and reducing the system’s complexity. Unlike the commonly used structure in the DenseNet model design, the backbone network consists of a stem block and six phases of dense and transition blocks. Only the sixth phase does not use a transition block.

#### 3.1.1. Stem Block

**Figure 3:**
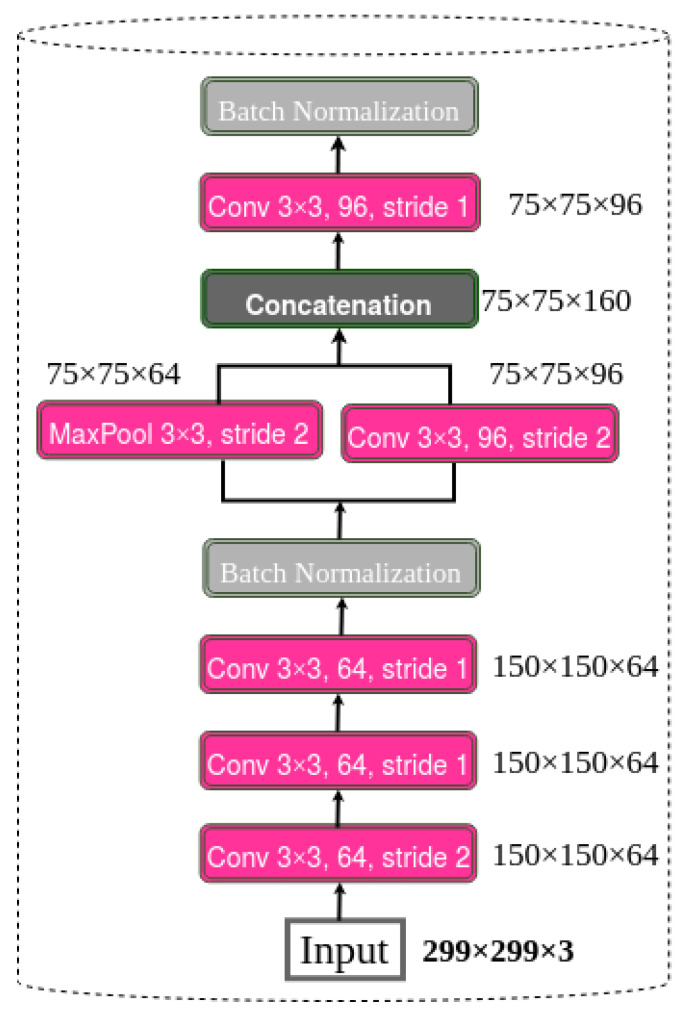
Structure of stem block.

The motivation behind the DenseNet-46 stem block design originates from the considerable success of the Inception-v4 structure [35] in small polyp detection. It is designed in the same manner before the first dense block, as depicted in Figure 3. Experimentally, the DenseNet-46 stem block can slightly reduce the information loss from input frames compared with the conventional structure of DenseNet, which includes a 7 × 7 convolutional layer and a 3 × 3 max pooling before the first dense block. The redesigned stem block can also reinforce the DC-SSDNet network’s ability for feature extraction with less computational cost. However, the DenseNet-46 stem block structure excerpted from the asymmetric convolution kernel structure used in Inception-v4 reduces the model complexity while maintaining a small loss of feature information.

#### 3.1.2. Dense Block

**Figure 4:**
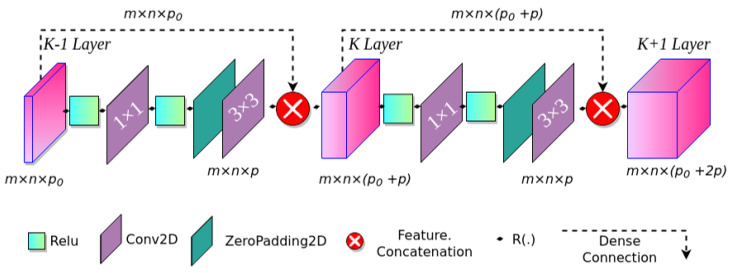
Structure of dense block.

The dense block is the basic unit of the compact DenseNet-46 structure, as shown in Figure 4. We denote the feature maps of the K − 1 layer as $m\times n\times p_{0}$, where m and n represent the width and height of feature maps, and $p_{0}$ means the number of channels. R(·) represents a nonlinear transformation consisting of a rectified linear unit (Relu) as an activation function, a 1×1 convolution layer, and a 3×3 convolution layer. It changes the number of channels to k (k = 32) without altering the size of the feature maps. To reduce the number of channels, we used the 1×1 convolution operation, and we adopted the 3×3 convolution operation for feature restructuring and to improve the network performance. Each dense block reduces the redundancy of dense connectivity represented by the long dashed arrow, in which feature maps of the K − 1 layer are connected directly to those of the K layer and then make a concatenation with the output of R(·), thus resulting in $m\times n\times (p_{0}+p)$. The output of the K + 1 layer is $m\times n\times (p_{0}+2p)$. Previous studies based on DenseNet used a fixed number of dense blocks (4 dense blocks in all DenseNet architectures) to keep the same scale of outputs. They also added new layers to each DenseNet network’s dense blocks to increase framework depth. A connectivity increase may decrease network performance due to contextual and semantic information redundancy. For this reason, the proposed DenseNet-46 backbone structure adds two additional dense blocks to increase the network depth rather than increasing the connections of each dense block. The DenseNet-46 backbone structure used in this work has two base blocks in dense blocks (1), three in dense blocks (2), three in dense blocks (3), three in dense blocks (4), two in dense blocks (5), and two in dense blocks (6).

#### 3.1.3. Transition Block

**Figure 5:**
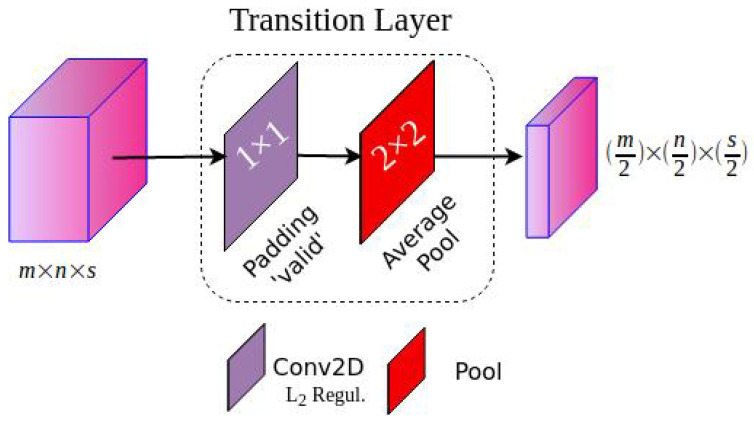
Structure of transition block.

After several dense connections, the number of feature maps of the DenseNet-46 grows dramatically. Consistent with the original DenseNet, we designed transition layer blocks to reduce the dimension of previous dense blocks’ features, as depicted in Figure 5. The transition block includes a 1×1 convolution layer with valid padding to reduce the number of channels of the previous layers. Then, it performs a 2 × 2 average pooling layer to decrease half of the feature map sizes. In the last phase, the transition block is not used to not reduce the final feature map resolution for further modification related to the SSD output requirements.

#### 3.1.4. Growth Rate

Table 1 shows the details of the compact DenseNet-46 backbone. The growth rate of the DenseNet denoted as ’k’ is referred to as the number of 3×3 convolution kernels in each dense block. It changes the number of channels of the input feature maps in each dense block. Since each dense block is concatenating to its previous ones, the succeeding layer channels grow by k after each dense block. The number of base blocks in the dense block, which consists of 4k 1 × 1 convolutions and 1k 3 × 3 convolutions, varies depending on the dense block location. It is well known an increase in growth rate gives good performance due to the large amount of information circulating in the network. This assumption is not always verified as it depends on the context environment, and the system complexity will also increase. In the DenseNet-64 base model used in this proposal, we utilized three settings: k = 16, k = 32, and k = 48. The growth rate choice was validated experimentally.

### 3.2. Multiscale Feature Pyramid Network

Several feature layers (Conv6_2, Conv7_2, Conv8_2, and Conv9_2) are added to the original SSD via the base network’s end (VGG16) for object classification and location regression from multiple feature maps at different depths. Inspired by the pyramidal feature hierarchy, the proposed approach adopts the SSD network structure [12]. However, the original backbone network VGG-16 of SSD is replaced with DenseNet-46 to enhance the feature extraction ability of the SSD model and address the problem of scale variations in detecting small polyp regions. The DC-SSDNet network uses the output of each transition block as the input of the multiscale feature pyramidal except for the sixth phase, to keep the final feature map resolution not reduced. Convolutional layers are used to perform pyramidal feature maps for small polyp detection but with different configurations. Consistent with the SSD model, the final feature layers have target sizes of 38×38, 19×19, 10×10, 5×5, 3×3, and 1×1.

## 4. Experiments

### 4.1. Datasets and Experimental Environments

**Figure 6:**
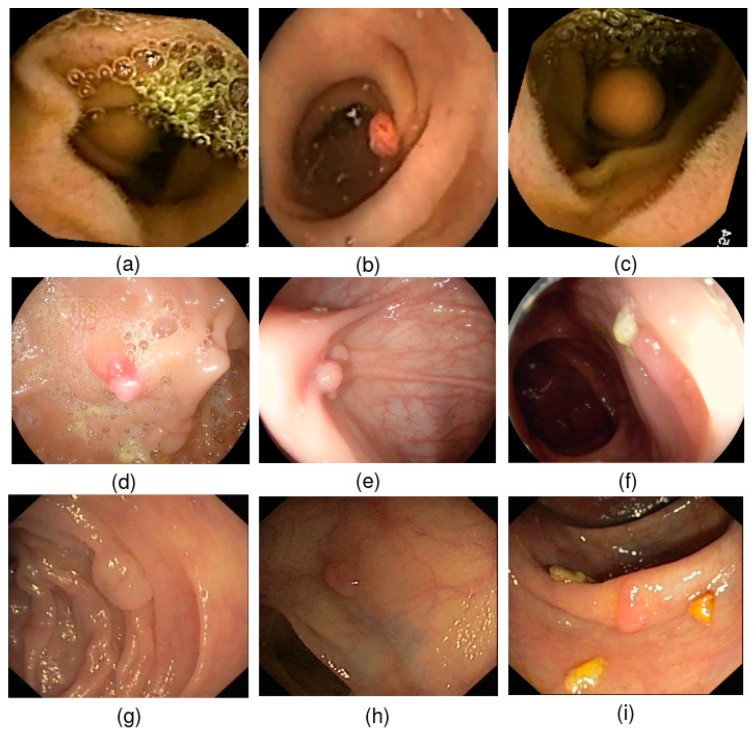
Example of samples: (**a**–**c**) WCE polyp images; (**d**–**f**) Etis-Larib polyp images; (**g**–**i**) CVC- ClinicDB polyp images.

The WCE dataset is from PillCam©COLON 2 polyps [36]. It consists of 120 polyps and 181 normals images collected from one patient’s VCE test with a resolution of 256 pixels × 256 pixels, as shown in Figure 6. A preprocessing stage was performed to avoid overfitting and increase the training dataset size. Hence, the regenerated dataset comprised 1250 polyp patches and 1864 normal ones. Two highly qualified experts manually labeled and annotated frames as positive and negative samples. To provide ground truths, they defined binary masks corresponding to the polyp regions covered. To meet the needs of polyp detection tools, the bounding boxes of polyp regions were delimited using the specialists’ ground-truth mask using a graphical image annotation tool to label objects’ bounding boxes in images (LabelImg). Then, experts corrected them. The second dataset was the popular CVC-ClinicDB [37]. In this regard, researchers reviewed 25 colonoscopy videos to choose at least 29 sequences with at least one polyp region and selected a set of frames for each of them. The CVC-ClinicDB dataset contains 612 polyp images with a resolution of 384 × 288. The specialists manually defined masks for the polyp-covered regions in each image to provide the ground truths. Then, they were drawn based on the ground truth provided by the specialists using advanced medical annotation tools. To confirm the proposed model’s credibility, we used the annotated ETIS-Larib dataset [38] in which 34 colonoscopy videos produced 196 polyp images of various shapes and sizes, as shown in Figure 6. Skilled experts annotate the ETIS-Larib dataset ground truths. The colonoscopy images were from the MICCAI 2015 subchallenge on the automatic polyp detection task. Polyps’ images were rescaled to 299 × 299 pixels. We divided the data into 70% for training, 10% for validation, and 20% for model testing. We employed a fivefold cross-validation to validate the model’s state and convergence after each epoch. The validation data step automatically adjusted the iterations and the learning rate. The model adopted a validation set according to the five group performances in the models. Finally, the performances were averaged across the splits to calculate the mean average precision measure.

In this study, we performed the training and testing phases using the Colab Pro Plus solution provided by Google, with a maximum RAM of 52 Gb and a disk of 166.83 Gb. The CUDA 8.0.61-1, CuDNN6.0, Keras 2.1.0, Python 3.7, protobuf 3.20.*, h5py 2.10.0, NumPy 1.16.3, TensorFlow 1.14, TensorFlow-GPU 1.14, OpenCV-python, scikit-learn, scikit-image, tqdm, beautifulsoup4, lxml, html5lib, bs4, ipykernel, and OpenCV 3.1 packages were used to implement the algorithm. For all used datasets, an aspect ratio between one and two was adopted depending on the small polyp regions’ true bounding boxes.

### 4.2. Training

**Figure 7:**
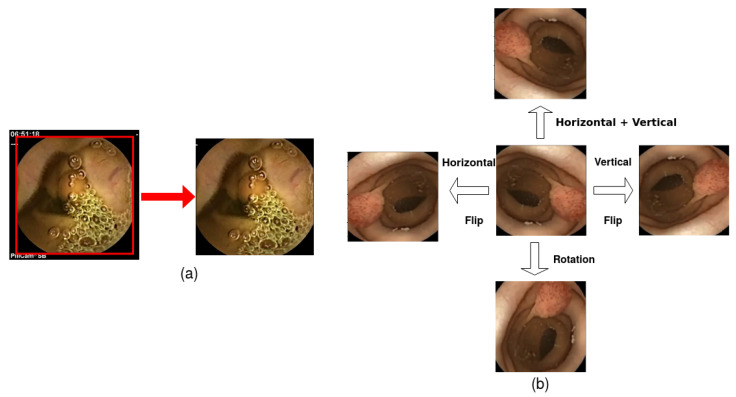
A flow-chart of the preprocessing steps: (**a**) acquiring the region of interest (ROI) of the WCE polyp image (**b**); sample of image from the WCE dataset with geometric transformation.

Many training hyperparameters were adjusted to strengthen the DC-SSDNet capability for detecting polyp abnormalities in WCE and colonoscopy frames as shown in Table 2. The presence of black regions in WCE images may impair detection performance and lengthen computation time. Hence, the original capsule endoscopy image was reduced to a center square-shaped image in the peripheral area as a first preprocessing step in order to remove unwanted black regions. We used a batch size of 32 and rescaled the input images to (299 × 299 × 3). The model employed 100 training epochs and 500 steps per epoch. The motivation behind the data augmentation was a lack of data for the WCE classification and detection tasks. Moreover, data access was tightly restricted owing to privacy concerns. In this regard, we applied popular augmentation methods used in recent studies [10,35]. We applied geometric methods which altered the geometry of the resulted region-of-interest (ROI) image as a second preprocessing step, by mapping the individual pixel values to new destinations. To perform data augmentation techniques, the flipping approach was examined in order to mirror the ROI WCE frames across their vertical and horizontal axes at first, then across both in the second pass. Finally, we rotated the ROI WCE images by $270^{\circ }$ about their center, as shown in Figure 7.

We trained the proposed model from scratch to not rely on any model pretrained on classification tasks to initialize the network, as commonly known visual purposes of classification and detection are distinct. For training DC-SSDNet, we used adaptive moment estimation (Adam) as the optimizer (beta_1 of 0.9, beta_2 of 0.999, and an epsilon of 1 × 10^−8^). The learning rate was initially set to 0.0001, and the learning rate decay policy differed slightly from the original SSD with a drop of 0.5 and an epoch drop of 10, which allowed the network to converge by controlling its learning rate. If the epoch number was less than 80, the learning rate was 0.0001, 0.00001 if the epoch number was less than 100, and 0.000001 otherwise. Recent state-of-the-art approaches targeting polyp detection were investigated in this study to compare them with the proposed model [10]. We used a loss function consistent with that used in the traditional SSD [12]. The cross-validation method adjusted the α parameter to one and the neg_pos_ratio to three. More information about SSD_Loss can be found in [16]. The proposal evaluated the detection performance with the most used metrics in the field, the mean average precision (mAP), the number of frames per second (FPS), and other indicators.

### 4.3. Evaluation Indexes

This study evaluated polyp detection performance using the mean average precision (mAP) and frames per second (FPS), which are well-known in target object detection. The mAP represents the average of all object categories’ average precision (AP), expressed as:

(1) $$mAP=\frac{\sum_{s=1}^{S}AveP\left(s\right)}{S}$$

where S denotes the number of queries in the set and s denotes the average precision query.

The precision indicator represents a measure of exactness and the recall a measure of completeness. The expected overlap between the ground-truth bounding box annotated by the experts and the predicted one produced by the network expresses the intersection over union (IoU). The following equations formulate the precision, recall, and F1-score indicators as follows:

(2) $$Precision=\frac{TP}{TP+FP}\;Recall=\frac{TP}{TP+FN}$$

(3) $$F-measure/F1-score=\frac{\left(2\times Recall\times Precision\right)}{Recall+Precision}$$

where TP represents the true positives with an IoU greater than 0.5, FP represents the false positives, and FN represents the false negatives. The frames per second (FPS) metric measures the detection speed and denotes the number of frames sent per second. A detailed evaluation metric of the model’s performance is provided in the work of [10].

### 4.4. Results and Discussion

#### 4.4.1. Ablation Studies

On the WCE, CVC-ClinicDB, and ETIS-Larib colonoscopy datasets, we performed an ablation study to investigate the impact of each component of the DC-SSDNet detector on performance. Table 3 shows different model settings using the compact DenseNet-46 as a backbone, where the training was conducted on the WCE images, the CVC-ClinicDB+ETIS-Larib joint training sets, and tested on the WCE, CVC-ClinicDB, and ETIS-Larib test sets. Using three values of the growth rate K, the performance was 81.96% when K = 16 and improved to 85.41% mAP at K = 32. The use of a small growth rate produced better results for K = 32, and a larger K = 48 could also provide a better model performance according to the DF-SSD as it showed a smaller mean AP of 84.54% than K = 32. It is highly recommended not to set a higher growth rate to reduce network complexity and computing costs. The growth rate of DC-SSDNet was set to 32. Table 3 demonstrates that the stem block improved the model’s mAP performance by 7.78% (89.74% vs. 81.96%) at K = 16, 8.55% (93.96% vs. 85.41%) at K = 32, and 7.08% (91.62% vs. 84.54%) at K = 48 on the WCE dataset. The results proved the relative importance of stem blocks in preserving information in the original input image and contributing to small polyp detection. Then, we evaluated transition pooling techniques (average pooling and max pooling) and report their influence on the proposed system’s performance mAP (%). We can see from Table 3 that an average pooling on the WCE test set obtained a higher mAP of 93.96% compared with max pooling (90.56% mAP) using the stem block at K = 32. Consistent with the traditional DenseNet, we used the average pooling layer to decrease the resolution of the feature maps. As reported in Table 3 (row 5, row 6, and row 8), without batch normalization for each conv layer, the proposed approach obtained better results (mAP of 85.41%, 90.56%, and 93.96%) at K = 32, whereas the mAP was 83.54% and 84.97% when utilizing batch normalization on the WCE training and test sets at K = 32 and K = 48, respectively. The ETIS-Larib and CVC-ClinicDB test sets in Table 3 (rows 11–20 and 21–30) complement the WCE image results and illustrate the efficacy of the stem block and growth rate parameters in emphasizing more salient aspects for small polyp identification.

#### 4.4.2. SSD Results on WCE and Colonoscopy Datasets

We trained the detector-based SSDs on the WCE, CVC-ClinicDB, and ETIS-Larib combined training set and tested on each dataset separately as depicted from Table 4 and Table 5. SSD300 with VGG16 (as a backbone) showed that the WCE, CVC-ClinicDB, and ETIS-Larib test sets had mAPs of 77.2%, 74.5%, and 74.22%, respectively. Table 4 and Table 5 (rows 5–6) prove that the FSSD300 and FSSD500 models showed gains of 12.58% and 9.25%, 12.76% and 9.16%, and 12.18% and 11.47% in terms of mAP compared to the original SSD. DenseNet-S-32-1 replaces VGGNet as the backbone network on the DF-SSD300. Thus, for the WCE, CVC-CLinicDB, and Etis-Larib data sets, it outperformed the FSSD300 model by 1.46%, 2.66%, and 0.54% for the mAP with 91.24% vs. 89.78%, 89.92% vs. 87.26%, and 86.84% vs. 86.3%, respectively. To incorporate contextual and semantic information, the L_SSD model in Table 4 and Table 5 (row 8) replaced the VGG16 network with the ResNet-101 backbone showing an improvement in terms of mAP compared to the FSSD model for all employed datasets. The DF-SSD300 algorithm outperformed the L_SSD algorithm in terms of mAP with a gain of 1.26 points (91.24 % vs. 89.98%), 1.74 points (89.92 % vs. 88.18%) on the WCE and CVC-ClinicDB test sets, respectively, due to the power of DenseNet in feature reuse and extraction abilities. However, it showed a slight drop in mAP on the Etis-Larib test set. The proposed DC-SSDNet model surpassed the DF-SSD300, MP-FSSD, and Hyb-SSDNet networks by 2.72 points (93.96% vs. 91.24%), 0.56 points (93.96% vs. 93.4%), and 0.67 points (93.96% vs. 93.29%) on the WCE dataset. The DC-SSDNet framework achieved a 32.5 FPS real-time detection, compared to 11.6 FPS, 17.4 FPS, and 40 FPS for the DF-SSD300, DSOD300, and L-SSD, respectively.

#### 4.4.3. Comparison with Existing Detection Methods

We compared the performance of the proposed DC-SSDNet approach to the most prominent networks in the literature-based SSDs models, YOLOv3 and Faster R-CNN, as shown in Table 6. For further comparing DC-SSDNet with previous studies’ target polyp detection, we trained the model on the joint ETIS-Larib and CVC-ClinicDB training sets and evaluated it on the publicly accessible ETIS-Larib dataset. The DC-SSDNet model achieved promising results or similar metrics in the worst cases on the three employed test sets compared to other state-of-art approaches. One of the main reasons for the difference in results was that the nature, texture, and lighting conditions of the WCE and colonoscopy images change inside the GI tract. Following a series of improvements to the initial SSD model, DC-SSDNet achieved an mAP of 93.96% utilizing only the WCE for both training and test sets, and outperforming other methods on the CVC-ClinicDB and Etis-Larib test sets by 92.24% and 90.86%, respectively. Due to the fact the WCE images were gathered from a single patient’s VCE test, and CVC-ClinicDB images were acquired from 25 colonoscopy recordings to choose at least 29 sequences with at least one polyp area and a series of frames for each of them, even if the WCE was accurately divided into training, validation, and testing sets, there would be some overlap. As a result, the network might have been familiar with certain hard cases. Moreover, it showed superior performance compared to the YOLOv3 model [21] and close to that of Hyb-SSDNet [35]. DC-SSDNet yielded good results in terms of mAP while maintaining the computational cost and a small speed drop.

#### 4.4.4. Visualization

**Figure 8:**
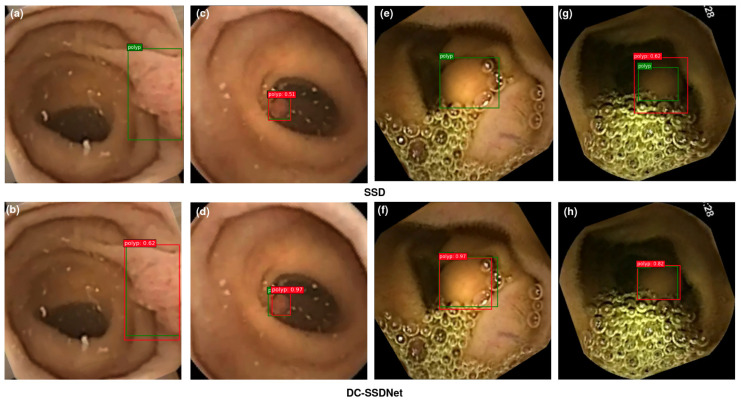
On the WCE test set, visualization results comparing SSD300 (**a**,**c**,**e**,**g**) and the proposed DC-SSDNet network (**b**,**d**,**f**,**h**). True bounding boxes with IoU of 0.5 or greater are drawn in green, whereas predicted bounding boxes are in red.

**Figure 9:**
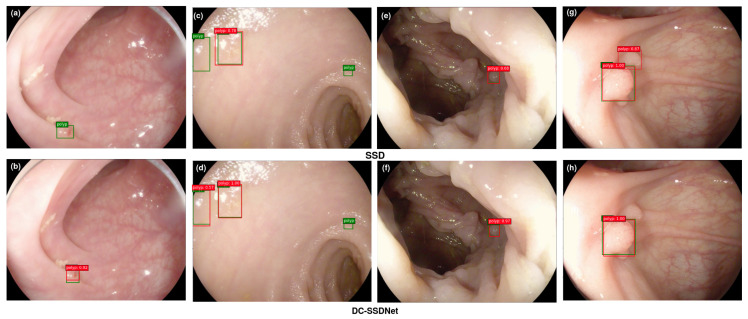
On the Etis-Larib test set, visualization results comparing SSD300 (**a**,**c**,**e**,**g**) and the proposed DC-SSDNet network (**b**,**d**,**f**,**h**). True bounding boxes with IoU of 0.5 or greater are drawn in green, whereas predicted bounding boxes are in red.

**Figure 10:**
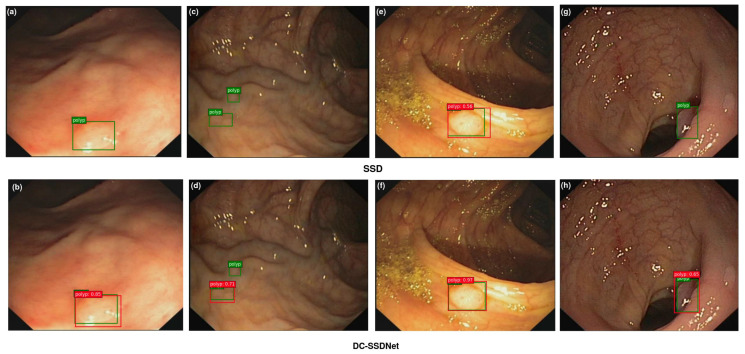
On the CVC-ClinicDB test set, visualization results comparing SSD300 (**a**,**c**,**e**,**g**) and the proposed DC-SSDNet network (**b**,**d**,**f**,**h**). True bounding boxes with IoU of 0.5 or greater are drawn in green, whereas predicted bounding boxes are in red.

The main objective of this study was to show the impact of the condensed model in highlighting small polyp regions for the detection and localization tasks on the WCE, Etis-Larib, and CVC-ClinicDB test sets. Polyp detection aims at selecting polyp areas and ignoring normal parts, feces, artifacts, and water jet sprays to clean the colon. Some examples of polyp detection of the SSD model and DC-SSDNet network on the employed datasets are depicted in Figure 8, Figure 9 and Figure 10. Compared to DC-SSDNet (Figure 8b,f, Figure 9b,d, and Figure 10b,d,h), Figure 8a,e, Figure 9a,c, and Figure 10a,c,g show false negative results in which the conventional SSD failed to detect small and flat polyp regions. The polyp region appeared similar to the surrounding normal mucosa as well the existence of food, air bubbles, and other debris may have hindered its localization process. Polyp areas showed variations related to color, texture, size, and shape and required more compact models to limit the number of false negatives and avoid missed detection. DC-SSDNet performed feature reuse by directly connecting shallower and deeper layers to distinguish polyp edge areas from normal ones. Figure 9g shows a false positive case of the SSD model on the ETIS-Larib test set with an error in detecting the polyp region while it was not there, whereas Figure 8b,f, Figure 9b,d,g, and Figure 10b,d,h show the correct identifications of the DC-SSDNet network. Besides small polyp detection, the proposed DC-SSDNet network achieved promising performance even for detecting large polyps on the WCE, CVC-ClinicDB, and ETIS-Larib test sets; see Figure 8b. With the presence of multiple polyps in one frame and a low contrast between the polyps and the background regions, both SSD and DC-SSDNet missed localizing small polyp regions, as illustrated in Figure 9c,d and Figure 10c,d. In these cases, the problem was most likely one of contrast since the polyp was oversaturated. As a result, the F1 score decreased significantly. Although DC-SSDNet produced promising mAP results, it failed to detect some small polyp instances that seemed similar to portions of the colon due to lighting and contrast, resulting in a misleading bounding box and lowering the mAP and F1 scores. Even endoscopists may miss some small parts that cannot be detected by the naked eye or that are hidden from view behind a fold. However, the proposed DC-SSDNet model proved its efficiency in localizing small polyp regions at a fast detection speed while ensuring precision.

## 5. Conclusions

This work suggested an improved SSD detector based on a redesigned DenseNet (DC-SSDNet) emphasizing polyp detection in the WCE, Etis-Larib, and CVC-ClinicDB datasets. Many researchers in the field use their own datasets due to medical imaging ethics and the lack of publicly available and annotated WCE polyp datasets. Thus, their results may suffer from subjectivity. The DC-SSDNet network aimed to exploit the potential of feature reuse as opposed to relearning features in later layers. The proposed approach used stacked dense and transition blocks instead of simple convolution layers with a capacity of the SSD network to be more compact while going deeper, yielding condensed models that were easy to train and highly parameter-efficient. The compact DenseNet-46 compressed unnecessary convolution layers of each dense block to reduce the amount of feature redundancy, resulting in fewer overall parameters and faster training times. The small polyp areas’ visual appearance was modeled by directly connecting layers throughout the network and generating novel pyramidal feature maps. While training data from scratch, the DC-SSDNet detector achieved comparable, if not superior, performance in mAP compared to other state-of-the-art pretrained models with real-time processing speed on the WCE and public datasets (CVC-ClinicDB and Etis-Larib). Artifacts and other factors may degrade performance and negatively affect the detection process. We will conduct further studies in the future around this shortcoming while maintaining the smaller running time. Rather than proposing polyp identification tasks on WCE and colonoscopy frames, future studies will investigate hybrid architectures to present a unique detection method for video colonoscopy. Furthermore, it would be interesting to use a hybrid 2D/3D architecture and assess its performance by employing other promising backbones.

## Figures and Tables

**Table 1:** Compact DenseNet-46 architecture (growth rate k = 32) in each dense block.

Phase	Layer	Setting	Output Size (Input 299 × 299 × 3)
	Stem block		75×75×96
Phase 1	Dense block (1)	1×1Conv3×3Conv⌊Pad−Valid⌋×2	75×75×160
	Transition block (1)	1×1Conv⌊Pad−valid⌋	75×75×160
		2×2AveragePool⌊stride2⌋	38×38×160
Phase 2	Dense block (2)	1×1Conv3×3Conv⌊Pad−Valid⌋×3	38×38×256
	Transition block (2)	1×1Conv⌊Pad−valid⌋	38×38×256
		2×2AveragePool⌊stride2⌋	19×19×256
Phase 3	Dense block (3)	1×1Conv3×3Conv⌊Pad−Valid⌋×3	19×19×352
	Transition block (3)	1×1Conv⌊Pad−valid⌋	19×19×352
		2×2AveragePool⌊stride2⌋	10×10×352
Phase 4	Dense block (4)	1×1Conv3×3Conv⌊Pad−Valid⌋×3	10×10×448
	Transition block (4)	1×1Conv⌊Pad−valid⌋	10×10×448
		2×2AveragePool⌊stride2⌋	5×5×448
Phase 5	Dense block (5)	1×1Conv3×3Conv⌊Pad−Valid⌋×2	5×5×512
	Transition block (5)	1×1Conv⌊Pad−valid⌋	5×5×512
		2×2AveragePool⌊stride2⌋	3×3×512
Phase-6	Dense block (6)	1×1Conv3×3Conv⌊Pad−Valid⌋×2	3×3×576

**Table 2:** Hyperparameter settings of the densely connected single-shot multibox detector (DC-SSDNet).

Hyperparameters	Values
Optimizer	Adam
beta_1	0.9
beta_2	0.999
epsilon	1 × 10^−8^
Initial learning rate	0.0001
Learning rate decay drop factor	0.5
Epoch drop factor	10
	epoch < 80: 0.0001
Learning rate	epoch < 100: 0.00001
	0.000001 otherwise
α parameter	1
neg_pos_ratio	3
Batch size	32
Training epochs	100
Steps per epoch	500
Aspect ratio	1–2

**Table 3:** Results of an ablation study on the WCE and colonoscopy datasets. BN denotes the addition of a batch normalization layer to each convolution layer. K refers to the growth rate. The stem block represents the front layers of the DenseNet-46 backbone. The transition pool is either an average pooling layer or a max pooling layer. The mAP represents the mean average precision on the WCE, Etis-Larib, and CVC-ClinicDB test sets.

Training Data	Test Data	Stem Block	K	BN	Transition Pool	mAP (%)
		×	16	×	Average	81.96
		×	32	✓	Max	83.54
		✓	48	✓	Average	91.62
		×	48	✓	Max	84.97
WCE	WCE	×	32	×	Average	85.41
		✓	32	×	Max	90.56
		✓	16	×	Max	88.16
		✓	32	×	Average	**93.96**
		✓	16	×	Average	89.74
		×	48	×	Average	84.54
		×	16	×	Average	80.68
		×	32		Max	81.32
		✓	48	✓	Average	89.22
		×	48	✓	Max	83.14
CVC-ClinicDB		×	32	×	Average	83.75
joint	CVC-CLinicDB	✓	32	×	Max	88.09
Etis-Larib		✓	16	×	Max	87.09
		✓	32	×	Average	**92.24**
		✓	16	×	Average	89.36
		×	48	×	Average	84.77
		×	16	×	Average	79.98
		×	32	✓	Max	81
		✓	48	✓	Average	89.46
		×	48	✓	Max	83.98
CVC-ClinicDB		×	32	×	Average	84.52
joint	Etis-Larib	✓	32	×	Max	87.55
Etis-Larib		✓	16	×	Max	87.14
		✓	32	×	Average	**90.86**
		✓	16	×	Average	89.34
		×	48	×	Average	82.72

**Table 4:** SSD comparison with previously reported approaches based on the WCE test set. Pretrain denotes a pretrained backbone to initialize the model, as opposed to starting from scratch. The Google Colab pro+ GPU was used to assess the speed (FPS) and performance (mAP).

Training Data	Methods	Backbone	Input Size	Pretrain	FPS	mAP@0.5 (%)
	SSD300	VGG16	300 × 300 × 3	✓	46	77.2
	SSD300	ResNet-101	300 × 300 × 3	✓	47.3	81.65
	SSD500	VGG16	300 × 300 × 3	✓	19	79.45
	SSD500	ResNet-101	300 × 300 × 3	✓	20	84.95
WCE	FSSD300	VGG16	300 × 300 × 3	✓	65.9	89.78
	FSSD500	VGG16	500 × 500 × 3	✓	69.6	88.71
	DF-SSD300 [16]	DenseNet-S-32-1	300 × 300 × 3	×	11.6	91.24
	L_SSD [39]	ResNet-101	224 × 224 × 3	✓	40	89.98
	MP-FSSD [10]	VGG16	300 × 300 × 3	✓	62.57	93.4
	Hyb-SSDNet [35]	Inception v4	299 × 299 × 3	✓	44.5	93.29
	DSOD300 [40]	DS/64-192-48-1	300 × 300 × 3	×	17.4	91.70
	DC-SSDNet (ours)	DenseNet-46	299 × 299 × 3	×	32.5	**93.96**

**Table 5:** SSD comparison with previously reported approaches based on CVC-ClinicDB and Etis-Larib test sets. Pretrain denotes a pretrained backbone to initialize the model, as opposed to starting from scratch. The Google Colab pro+ GPU was used to assess the speed (FPS) and performance (mAP).

Training Data	Methods	Backbone	Input Size	Pretrain	FPS	mAP@0.5 (%)	
						CVC-ClinicDB	ETIS-Larib
	SSD300	VGG16	300 × 300 × 3	✓	46	74.5	74.12
	SSD300	ResNet-101	300 × 300 × 3	✓	47.3	78.85	75.73
CVC-ClinicDB	SSD500	VGG16	500 × 500 × 3	✓	19	78.38	75.45
joint	SSD500	ResNet-101	500 × 500 × 3	✓	20	82.74	80.14
ETIS-Larib	FSSD300	VGG16	300 × 300 × 3	✓	65.9	87.26	86.3
	FSSD500	VGG16	500 × 500 × 3	✓	69.6	87.54	86.92
	DF-SSD300 [16]	DenseNet-S-32-1	300 × 300 × 3	×	11.6	89.92	86.84
	L_SSD [39]	ResNet-101	224 × 224 × 3	✓	40	88.18	87.23
	MP-FSSD [10]	VGG16	300 × 300 × 3	✓	62.57	89.82	90
	Hyb-SSDNet [35]	Inception v4	299 × 299 × 3	✓	44.5	91.93	91.10
	DSOD300 [40]	DS/64-192-48-1	300 × 300 × 3	×	17.4	90	89.3
	DC-SSDNet (ours)	DenseNet-46	299 × 299 × 3	×	32.5	**92.24**	**90.86**

**Table 6:** Results of the WCE or the colonoscopy test with an IoU greater than 0.5 and a batch size of 1.

Training Dataset	Methods	Testing Dataset	Backbone Network	Pretrain	Input Size	Prec (%)	Recall (%)	F1 Score (%)
WCE	DC-SSDNet (ours)	WCE	DenseNet-46	×	299×299	93.96%	90.82%	90.7%
CVC-ClinicDB + ETIS-Larib	DC-SSDNet (ours)	CVC-ClinicDB	DenseNet-46	×	299×299	92.24%	91%	88.40%
CVC-ClinicDB + ETIS-Larib	DC-SSDNet (ours)	ETIS-Larib	DenseNet-46	×	299×299	90.86%	90.4%	89.12%
CVC-ClinicDB + ETIS-Larib	Shin et al., 2018 [2]	ETIS-Larib	Inception ResNet	✓	768×576	92.2%	69.7%	79.4%
ETIS-Larib+CVC-ClinicDB	Souaidi et al., 2022 [35]	ETIS-Larib	Inception v4	✓	299×299	91.10%	87%	89%
SUN+ PICCOLO+ CVC-ClinicDB	Ishak et al., 2021 [21]	ETIS-Larib	YOLOv3	✓	448×448	90.61%	91.04%	90.82%
WCE +CVC-ClinicDB	Souaidi et al., 2022 [10]	ETIS-Larib	VGG16	✓	300×300	90.02%	×	×
CVC-ClinicDB	Liu et al., 2021 [41]	ETIS-Larib	ResNet-101	✓	384×288	77.80%	87.50%	82.40%
GIANA 2017	Wang et al., 2019 [42]	ETIS-Larib	AFP-Net(VGG16)	✓	1225×996	88.89%	80.7%	84.63%
CVC-ClinicDB	Qadir et al., 2021 [43]	ETIS-Larib	ResNet34	✓	512×512	86.54%	86.12%	86.33%
CVC-ClinicDB	Pacal and Karaboga, 2021 [44]	ETIS-Larib	CSPDarkNet53	✓	384×288	91.62%	82.55%	86.85%
CVC-ClinicDB	Wang et al., 2019 [42]	ETIS-Larib	Faster R-CNN (VGG16)	×	224×224	88.89%	80.77%	84.63%
CVC-VideoClinicDB	Krenzer et al., 2019 [45]	CVC-VideoClinicDB	YOLOv5	×	574×500	73.21%	×	79.55%

## Data Availability

Publicly available datasets were analyzed in this study. The CVC-ClinicDB datasets are publicly available here: https://polyp.grand-challenge.org/CVCClinicDB/ (accessed on 23 January 2023). The ETIS-Larib dataset is publicly available here: https://polyp.grand-challenge.org/EtisLarib (accessed on 25 January 2023).

## Abbreviations

GI tract	Gastrointestinal tract
WCE	Wireless capsule endoscopy
MICCAI	Medical Image Computing and Computer Assisted Intervention
YOLO	You only look once
SSD	Single-shot multibox detector
R-CNN	Region-based convolutional neural network
R-FCN	Region-based fully convolutional network
DenseNet	Dense network
DCNN	Deep convolutional neural network
pool	pooling
ResNet	Residual network
FPN	Feature pyramid network
DSSD	Deconvolutional single-shot detector
ROI	Region of interest
Conv	Convolutional layer
DC-SSDNet	Densely connected single-shot multibox detector network
MaxPool	Max pooling
Pad	Padding
AveragePool	Average pooling
L2 Regul	L2 regularization
VCE	Video capsule endoscopy
Adam	Adaptive moment
neg-pos-ratio	negative positive ratio
mAP	Mean average precision
FPS	Frames per second
TP	True positive
FP	False positive
IoU	Intersection over union
FN	False negative
BN	Batch normalization
FSSD	Fusion single-shot multibox detector
